# LOVE OR OBLIGATION TO “SEE THEM THROUGH:” BURDENS AND GAINS IN CAREGIVING FOR CENTENARIANS

**DOI:** 10.1093/geroni/igac059.2702

**Published:** 2022-12-20

**Authors:** Eric Ngai Yin Shum, Bobo Hi Po Lau, Peter Martin, Joey Chung Yue Siu, Karen Siu Lan Cheung, Cecilia Lai Wan Chan, Grace Man Yee Chan, James Ka Hay Luk

**Affiliations:** Hong Kong Shue Yan University, Hong Kong, Hong Kong; Hong Kong Shue Yan University, Hong Kong, Hong Kong; Iowa State University, Ames, Iowa, United States; Caritas Institute of Higher Education, Hong Kong, Hong Kong, Hong Kong; The University of Hong Kong, Hong Kong, Hong Kong; The University of Hong Kong, Hong Kong, Hong Kong; The Hong Kong Council of Service, Hong Kong, Hong Kong; TWGHs Fung Yiu King Hospital (FYKH), Hospital Authority, Hong Kong, Hong Kong

## Abstract

In Hong Kong, the population of centenarians increased from about 3,000 in 2011 to over 10,000 in 2021. The growth of this population has led to challenges concerning how far family caregivers, who are usually older adults themselves, could care for their spouse or parents. In 2021, we launched the 2nd Hong Kong Centenarian Study and included the voices of family caregivers. Notwithstanding the increased difficulties of caregiving during COVID outbreaks, our interviews with 120 caregivers revealed low to moderate scores of caregiving burden and gains (measured by 4-items from the Zarit Burden Scale and 5-items from the Positive Aspects of Caregiving Scale). Female and older (aged 70 or above) caregivers reported more emotional distress, burden, and poorer self-rated health, while younger caregivers (less than 70 years old) sustained a wider social network. Financial stress was related to smaller social network size and more emotional distress. When being asked what sustained their motivation to care for their spouse or for their parents, “filial obligation to see them through” and “repaying for love” were answered as key motivators. Caregivers also derived pride and satisfaction from contributing to the remarkable longevity of their loved ones or from witnessing their loved ones recovering from life-threatening traumas (e.g., falls, hospitalization), but felt helpless when faced with escalating care needs due to their own deteriorating physical health and capacities. “Double-old caregiving” will become more common, and society will need to overhaul the care system to support these motivated families who have escalated care needs.

